# Infiltrating CD16^+^ Are Associated with a Reduction in Peripheral CD14^+^CD16^++^ Monocytes and Severe Forms of Lupus Nephritis

**DOI:** 10.1155/2016/9324315

**Published:** 2016-12-13

**Authors:** Anabel Barrera García, José A. Gómez-Puerta, Luis F. Arias, Catalina Burbano, Mauricio Restrepo, Adriana L. Vanegas, Carlos H. Muñoz, Mauricio Rojas, Luis A. González, Gloria Vásquez

**Affiliations:** ^1^Grupo de Inmunología Celular e Inmunogenética, GICIG, Universidad de Antioquía, Medellín, Colombia; ^2^Grupo de Reumatología, Universidad de Antioquía, Medellín, Colombia; ^3^Grupo de Patología Renal y Trasplante, Universidad de Antioquía, Medellín, Colombia

## Abstract

Our aim was to characterize glomerular monocytes (Mo) infiltration and to correlate them with peripheral circulating Mo subsets and severity of lupus nephritis (LN).* Methods*. We evaluated 48 LN biopsy samples from a referral hospital. Recognition of Mo cells was done using microscopic view and immunohistochemistry stain with CD14 and CD16. Based on the number of cells, we classified LN samples as low degree of diffuse infiltration (<5 cells) and high degree of diffuse infiltration (≥5 cells). Immunophenotyping of peripheral Mo subsets was done using flow cytometry.* Results*. Mean age was 34.0 ± 11.7 years and the mean SLEDAI was 17.5 ± 6.9. The most common SLE manifestations were proteinuria (91%) and hypocomplementemia (75%). Severe LN was found in 70% of patients (Class III, 27%; Class IV, 43%). Severe LN patients and patients with higher grade of CD16^+^ infiltration had lower levels of nonclassical (CD14^+^CD16^++^) Mo in peripheral blood.* Conclusions*. Our results might suggest that those patients with more severe forms of LN had a higher grade of CD14^+^CD16^+^ infiltration and lower peripheral levels of nonclassical (CD14^+^CD16^++^) Mo and might reflect a recruitment process in renal tissues. However, given the small sample, our results must be interpreted carefully.

## 1. Introduction

Systemic lupus erythematosus (SLE) is the most representative of autoimmune diseases being renal involvement one of the main features. Lupus nephritis (LN) is one of the hallmark features of SLE, seen in 40%–60% of patients at the moment of diagnosis, and is one of main causes of mortality among these patients [1]. Among 10 to 15% can develop an end stage renal disease during the follow-up and might require renal replacement therapies and/or kidney transplant [2, 3].

In the pathogenesis of SLE, innate immunity and immune complex had been widely evaluated but several cellular mechanisms have not been described in depth. Monocytes (Mo) are heterogeneous cells in their morphology, phenotype, and function. In humans, three different subpopulations of circulating Mo have been described. Those subsets are defined according to the expression of CD14 and CD16. In healthy subjects, 90% of Mo are CD14^++^CD16^−^ (classic Mo); 4% have the phenotype CD14^++^CD16^+^ (intermediate) and are also considered mature and can participate in the inflammatory response. The third subpopulation are CD14^+^CD16^++^ (nonclassic) that in comparison with other subpopulations of Mo are low producers of IL-10 [4].

Although the role of these subpopulations is controversial, it is well known that they can recognize immune complex through CD16, additionally can differentiate to diverse types of cells such as macrophages or dendritic cells (DC), and can polarize adaptive immune responses [5].

The main modifications of subpopulations of Mo cells have not been studied extensively in SLE patients. We described recently that CD14^+^CD16^++^ Mo are decreased in patients with active SLE and display changes in the molecules associated with their functions [6]. Those findings may suggest a potential role of these subpopulations in the pathogenesis of SLE and ultimately in kidney tissue lesions in LN.

There are enough “in vitro” evidences that a subpopulation of CD16 can differentiate to DC under the presence of GM-CSF, TGF-*β*, and IL-10, but without complete maturation. In that way, nonclassic CD16^+^ Mo might play a role as an important immunomodulator for innate and adaptive response in SLE participating in tissue damage, even before its differentiation to macrophages [7–10].

Some previous studies have investigated some subpopulations of Mo in murine models [11], but there is limited information in humans [12, 13]. This is explained in part for the differences between murine and human phagocytes, for the wide variability on Mo subpopulations, and for the lack of equivalence in translation studies among murine and humans models.

Pathogenic mechanisms of LN are not well understood. Some previous studies suggested that phagocytes CD14^+^CD16^+^ might participate in glomerular infiltration, interact with immune complex, and regulate some phases of inflammatory response including the process of antigen presentation [14].

Given the previous information and the lack of studies in patients with LN, our aim was to characterize glomerular Mo infiltration in kidney tissue and to correlate those infiltrates with peripheral blood levels of Mo, disease features, disease activity, and histological characteristics.

## 2. Material and Methods

### 2.1. Selection of Sample

We included 328 biopsy samples with diagnosis of LN by histopathology and immunofluorescence from clinical records of Pathology Department, Universidad de Antioquia. 241 out of 328 samples were done at Hospital San Vicente Fundación at Medellín, Colombia, from 2000 to 2011. Only those patients older than 18 years were included (*n* = 169). Finally, 48 tissue samples were classified as good enough to perform new analysis.

### 2.2. Histopathological Studies and Immunohistochemistry

Kidney biopsies were embedded in paraffin and sectioned at 4 *μ*m; then those samples were deparaffinized in xylene and rehydrated using graded concentrations of ethanol. Samples were stained with hematoxylin and eosin and then were examined under a light microscope. Tissue histopathologic state was classified according to ISN/RPS criteria. Patients with proliferative LN were grouped such as severe LN. Activity and chronicity indices of the histological appearance also were assessed based on National Institutes of Health scores. One observer (LA) with no prior knowledge of the clinical course examined renal tissue to establish the diagnosis by using standard pathological methods. Patients with renal involvement at SLE onset or with less than 1 year of disease duration were classified as early LN.

Different antibodies were used including CD14: Thermo Scientific, Rockford, Illinois, USA, number MA5-11394, dilution 1 : 40; CD16: Thermo Scientific, number MA5-11413, dilution 1 : 200; and CD68: Dako, Carpinteria, USA, code M0876, dilution 1 : 200. Those monoclonal antibodies were incubated at room temperature during 60 minutes. Endogenous peroxidase was blocked with hydrogen peroxide. Antigen retrieval was done with heat (steam) and tris-buffer with ethylenediaminetetraacetic acid at pH 7.0 for 30 minutes. The detection system used was based on polymer (Ultravision Quanto, Thermo Fisher Scientific, Fremont, CA, USA).

Recognition of Mo cells was done using microscopic view. Positive markers were quantified using the following parameters: If the stain was positive but with <2 cells together the stain was classified as “diffuse,” and the diffuse infiltrate was scored as follows: high degree of diffuse ≥5 cells or low degree of diffuse <5 cells. Whether quantification detects more than 2 cells together was classified as “aggregates.” Based on the number of cell counts aggregates were classified as grade 1, less than 5 cell counts; grade 2, five to 10 cell counts; and grade 3, more than 10 cell counts.

### 2.3. Immunophenotyping of Monocytes Subsets

Mo subpopulations were defined as three subsets based on previous reports in classic, (CD14^++^CD16^−^), nonclassic (CD14^+^CD16^++^), and intermediate (CD14^++^CD16^+^) Mo [15]. Briefly, 25 microliters of EDTA-anticoagulated peripheral blood was stained with anti-CD14-PerCP, anti-CD16-FITC, and anti-HLA-DR-PE or the isotype controls. Samples were mixed and incubated for 20 min at room temperature in darkness. Red cells were lysed with 250 *μ*L of OptiLyse and analyzed using the FACS Canto™ II (Becton Dickinson, San Jose, CA). Manual counting was compared with absolute counting. All patients gave informed consent for study. This study was approved by the Ethics Committee of the Universidad de Antioquia Medical Research Institute.

### 2.4. Statistical Analysis

Several variables were dichotomized for the analysis. Patients with proliferative LN were grouped as severe LN. Additionally patients were classified according to disease duration (early LN versus non-early) and according to degree of CD16^+^ infiltration (low or high level) as we mentioned above. Values are represented as means. Differences in means in normally distributed variables were analyzed using the parametric *t*-test, and variables without a normal distribution were assessed using Wilcoxon's test. The analysis was made using the IBM SPSS v22 statistical package.

## 3. Results

A total of 48 Mestizo patients with biopsy proven LN were enrolled for the study, of which 87% were females with a mean disease duration of 2.91 ± 5.6 years. Fifty-six percent of patients had early onset LN. Median age for the whole group was 34.0 ± 11.7 years. Proteinuria was the most frequent renal feature (91%), followed by hematuria (66%) and sterile pyuria (38%). The main clinical features are summarized in Table 1. Mean 24-hour proteinuria levels were 4.668 ± 3906 mg and mean glomerular filtration rate (GFR) was 74.2 ± 40.6 mL/min. As expected, proteinuria levels were significantly higher in patients with severe LN forms than in patients without severe forms (4.301 ± 917 versus 626 ± 280 mg, *p* = 0.018). No differences in GFR were found among groups.

The Mean Systemic Lupus Erythematosus Disease Activity Index (SLEDAI) score was 17.5 ± 6.9. We observed a high disease activity in most of the patients, in part explained by the specific weight of renal involvement (12 points).

### 3.1. Pathological Features

Proliferative glomerulonephritis (Class IV) was detected in 21 (43.8%) patients, Class III was detected in 13 (27%) of patients, and Class II and Class V LN were detected in six (12.5%) patients each. Only two patients were classified as Class I LN. We used activity and chronicity index proposed by Rovin [14] as our guide for the description of histological findings. Most common findings for acute lesion were cellular proliferation (83%) and karyorrhexis (25%). Tubular interstitial infiltrates were found in 71% of the cases. Arterial and arteriole abnormalities were found in a minority of patients.

Chronic changes were less common reported in renal biopsies. Tubular and glomerular fibrosis were found in 37% and 8% of patients, respectively. Other chronic changes were tubular atrophy in 37% of patients, basal membrane thickening in 27% of patients, arterial sclerosis in 10.4%, and arterial hyalinosis in 8.3% of patients. Histological findings are shown in Table 2.

We compared activity and chronicity indices scores among Class II and Class IV LN based on National Institutes of Health scores. Both acute and chronicity indices on kidney biopsies were significantly higher in Class IV LN than in Class III LN patients. Mean acute index was 4.1 ± 1.7 versus 9.6 ± 4.4, *p* = 0.001, for Classes III and IV, respectively, and mean chronicity index was 0.8 ± 2.0 versus 1.9 ± 2.0, *p* = 0.032, for Classes III and IV, respectively.

### 3.2. Characterization of Mo Infiltrates

**Figure 1:**
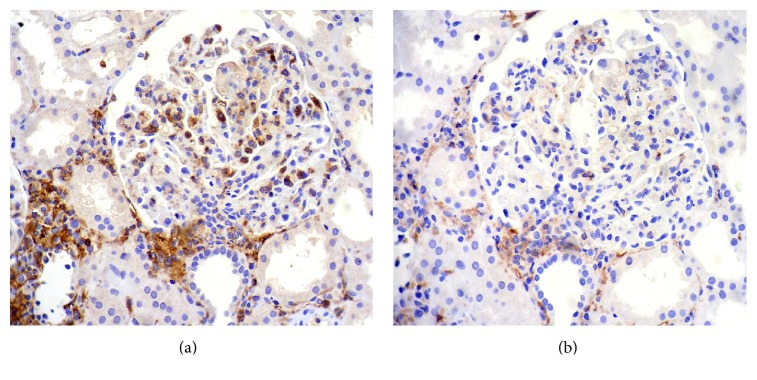
(a) Immunohistochemistry for CD16: abundant CD16^+^ cells in the glomerular tuft; these cells are also evidenced in the interstitium. (b) Immunohistochemistry for CD14: a glomerulus with few positive CD14^+^ cells. There are also some CD14^+^ cells in the interstitium. Both images, ×400.

Diffuse CD16^+^ cells were found in 25 biopsies and CD14^+^ cells in only sixteen biopsies. Representative pictures are shown in Figure 1 of the immunostainings for CD16^+^ and CD14 cells.

Patients with higher degree of CD16 diffuse infiltrates had a higher prevalence of hypocomplementemia (100% versus 0%, *p* = 0.028), higher but not significant levels of dsDNA antibodies, and higher disease activity (SLEDAI score ≥ 10). Five patients had CD16^+^ Mo aggregates. All patients who had grade 3 aggregates had Class IV LN in histologic classification. Grade 1 was observed in one patient with Class II and aggregates grade 2 was observed in one patient with Class III LN. With respect to CD14^+^ Mo cells, only accumulated grade 1 aggregates were observed in a patient with Class III LN.

### 3.3. Peripheral Mo Subsets

**Figure 2:**
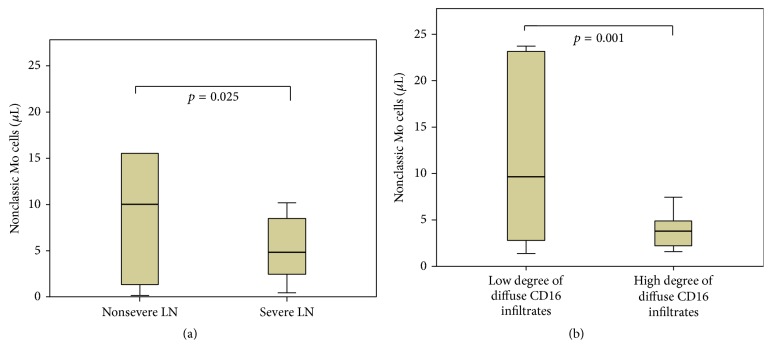
Peripheral blood levels of Mo subsets according to severity of LN (a) and degree of infiltrations by CD16^+^ cells (b).

Mo subsets around the time of kidney biopsy were available in 21 patients. Mean levels of absolute Mo were 497.63 ± 329 cells/*μ*L, classical Mo 459.46 ± 315.98 cells/*μ*L, intermediate Mo 17.33 ± 16.05 cells/*μ*L, and nonclassic Mo 10.09 ± 14.82 cells/*μ*L. When we classified patients in severe and nonsevere forms of LN, severe forms had lower levels of nonclassic Mo in peripheral blood (7.14 ± 7.22 versus 17.47 ± 25.32 cells/*μ*L, *p* = 0.025, Figure 2(a)). When we classified patients according to diffuse CD16^+^ infiltrates in high or low degree, patients with higher degree of infiltrates had lower levels of nonclassical Mo in peripheral blood (37.53 ± 21.98 versus 8.39 ± 5.70 cells/*μ*L, *p* = 0.001, Figure 2(b)).  Table 3 summarizes levels of different Mo subsets according to disease duration, severity of LN, and degree of infiltration.

At the same time, patients with higher degree of CD16^+^ infiltrates had higher but not significant activity index scores (5.46 ± 4.12 versus 4.70 ± 4.71, *p* = 0.363) and higher chronicity index scores (2.0 ± 2.72 versus 0.40 ± 0.51, *p* = 0.004). We did not find any association among subsets of Mo in peripheral blood with clinical characteristics, serological markers (including complement antibodies or anti-dsDNA) either with disease activity (SLEDAI).

## 4. Discussion

Mo and macrophages play an important role in murine models and human subjects with renal disease. Those cells have been involved in not only several processes such as renal injury and fibrosis but also several repair processes. We found that patients with more severe forms of LN (proliferative forms) have a higher grade of diffuse CD16^+^ Mo infiltrates. At the same time, severe forms had lower levels of peripheral nonclassical Mo and might reflect organ recruitment phenomenon of Mo cells in more severe forms of LN (proliferative Classes III and IV).

Linage cells from Mo/macrophages system constitute one of the most important cells involved in the inflammatory process of several renal diseases. Several studies have shown that the amounts of macrophages found in damaged tissues not only are bystanders of the inflammatory process but also play a role as active cells as occurs in some other in vitro studies [16]. Macrophages are distributed in tissues throughout the body and contribute to both homeostasis and disease. The recent increase in the availability of diverse surface markers for the determination of Mo/macrophages allowed the identification of different subpopulations in the kidney and other tissues. Mo subpopulations are functionally different. Some of these subpopulations have been related with a selective pattern of cytokine production [7, 8].

In vitro studies have demonstrated that macrophages can be polarized by activation with several cytokines. Activated and polarized cells have different functions. Initially, polarized cells were known as “classic” or “alternative” Mo. However, some other authors suggest that Mo should be labeled according to their phenotype (based on surface molecules) or according to their function [17–19]. The difference in the expression of lipopolysaccharide (LPS) receptor (CD14) and of Fc*γ* receptor III (CD16) has been used to distinguish various Mo subpopulations [4, 20].

We found a greater amount of diffuse infiltrations and aggregates of CD16^+^ cells in patients with more severe forms of LN. CD16^+^ are considered as nonclassic or intermediate Mo. It is well known that CD16^+^ cells produce TNF-*α* and interferon gamma (IFN*γ*) and participate in inflammatory process. Additionally, CD16^+^ express Class II molecules with a more efficient capacity for antigen presentation.

Previously, we observed that patients suffering from SLE had lower levels of CD16^+^ in peripheral blood, suggesting a differentiation process out of organs and cell recruitment in damaged organs [6]. Mo recruitment in inflammatory tissues could be explained in part by the active participation of Mo during all stages of organ damage process due to autoimmune phenomena. Mo participate during the clearance process of apoptotic bodies and immune complexes. In addition, Mo can induce the production of several cytokines involved in the pathogenesis of LN (for instance, IFN*γ*). Finally, Mo promote fibrosis and loss of epithelial cells and microvasculature [21, 22]. Given the association between chronicity index score and high degree of CD16^+^ infiltrates in our study, it is possible that CD16 Mo participate not only during early acute glomerular damage process but also during chronic fibrotic and microvascular processes as we mentioned previously.

Fractalkine (CX3CL1) and its receptor, CX3CR1, are known to mediate both cell adhesion and cell migration. In humans, CX3CR1 is expressed on Mo/macrophages and cytotoxic effector lymphocytes such as natural killer cells and cytotoxic T cells. In mice, its expression in circulating blood cells is restricted to Mo/macrophages and NK cells. Murine and human studies focused on the role of Mo in LN are limited. Using MRL/lpr murine model, Nakatani and colleagues [11] showed that fractalkine expression and CD16^+^ Mo infiltration increase with progression of the LN. In another study, a fractalkine antagonist was administered to MRL/lpr mice during early stages of LN [23]. MRL/lpr mice that received fractalkine antagonist exhibited significantly reduced glomerular hypercellularity, glomerulosclerosis, crescent formation, and vasculitis when compared with control mice.

Yoshimoto et al. [12] evaluated glomerular expression of fractalkine and CD16^+^ Mo in patients with LN. The authors found correlation of fractalkine glomerular expression with histopathologic activity index and proliferative forms of LN. Additionally, the amounts of infiltrating CD16^+^ Mo were higher in proliferative glomerular lesions of patients with SLE.

To the best our knowledge, this is the first study that correlated the levels of Mo in peripheral blood with Mo infiltrates in renal tissues. Our study suggested an inverse correlation among peripheral levels of nonclassic Mo and higher diffuse CD16^+^ infiltrative cells in glomerular tissue in patients with proliferative forms of LN.

Our study has some limitations. First, we analyzed a relative small number of patients with biopsy proven LN. However, despite this small sample we found significant differences among groups (severe and nonsevere) according to CD16^+^ infiltrates and subsets of Mo in peripheral blood. Given the nature of the study, we were not able to measure at the same time subsets of Mo in peripheral blood in all patients tested for Mo infiltrates on renal samples. Likewise, as our hospital is a reference center for systemic autoimmune diseases and predominantly received patients with limited access to health care, therefore our patients could be a selected sample of more severe SLE patients with LN. Finally, given that the majority of our patients were Mestizo, our results must be interpreted carefully and must be confirmed in other populations with different racial/ethnic distributions.

In conclusion our results in Mestizo patients with biopsy proven LN suggest that those patients with more severe forms of LN had lower peripheral levels of nonclassical Mo (CD14^+^CD16^++^). Reduction of these Mo subsets could be explained in part by a recruitment process in renal tissues. Given the small sample of the current study, our results must be interpreted carefully. Although the information about the role of Mo in LN is limited in clinical studies, animal model studies using fractalkine antagonist open a potential new target for the treatment of patients suffering from severe forms of LN. So far, there are no ongoing clinical trials evaluating the potential benefit of fractalkine antagonist in patients with LN.

## Figures and Tables

**Table 1:** General characteristics of 48 SLE patients with biopsy proven LN.

*Clinical characteristics*	
Mean age (years ± SD)	34.0 ± 11.7
Gender (female)	87%
Proteinuria	43 (91.5%)
Hematuria	31 (66.0%)
Arthritis	21 (44.7%)
Pyuria	18 (38.3%)
Urinary casts	16 (34.0%)
Pleuritis	11 (23.4%)
Alopecia	9 (19.1%)
Mouth ulcers	9 (19.1%)
Malar rash	8 (17.0%)
Pericarditis	6 (12.8%)
Vasculitis	6 (12.8%)
Fever	5 (10.6%)
Headache	1 (2.1%)
Seizures	1 (2.1%)
SLEDAI (SD)	17.5 ± 6.9

*Laboratory findings*	
Hypocomplementemia (either C3 or C4)	38 (80.8%)
dsDNA antibodies	27 (57.4%)
Leukopenia	8 (17%)
Thrombocytopenia	8 (17%)

*LN classification*	
Class I	2 (4.2%)
Class II	6 (12.5%)
Class III	13 (27.0%)
Class IV	21 (43.8%)
Class V	6 (12.5%)
Mean activity index score (SD)	5.7 ± 4.9
Mean chronicity index score (SD)	1.54 ± 2.2

**Table 2:** Histopathology characteristics in 48 patients with biopsy proven LN.

*Glomerular findings*	
Local necrosis	9 (18.7%)
Cellular proliferation	40 (83.3%)
Karyorrhexis	12 (25.0%)
Fibrinoid exudate	1 (2.1%)
Wire loop lesions	9 (18.7%)
Hyaline thrombosis	6 (12.5%)
Basal membrane thickening	13 (27.1%)
Fibrosis	4 (8.3%)

*Tubulointerstitial findings*	
Inflammatory infiltrate	34 (70.8%)
Edema	1 (2.1%)
Fibrosis	18 (37.5%)
Tubular atrophy	18 (37.5%)

*Artery and arterioles*	
Fibrinoid exudate	1 (2.1%)
Fibrinoid/platelet thrombus	1 (2.1%)
Necrosis	1 (2.1%)
Arterial sclerosis	5 (10.4%)
Arteriolar hyalinosis	4 (8.3%)

**(a):** 

	Early onset LN	Non-early onset LN	*p* value
Classic (CD14^++^CD16^−^)	411.53 ± 377.48	465.27 ± 138.48	0.021
Intermediate (CD14^++^CD16^−^)	14.35 ± 15.37	22.41 ± 18.17	0.488
Nonclassic (CD14^+^CD16^++^)	7.51 ± 7.38	15.32 ± 20.80	0.138

**(b):** 

	Severe LN	Nonsevere LN	*p* value
Classic (CD14^++^CD16^−^)	485.80 ± 345.81	393.63 ± 239.20	0.340
Intermediate (CD14^++^CD16^−^)	18.32 ± 18.11	14.84 ± 10.10	0.240
Nonclassic (CD14^+^CD16^++^)	7.14 ± 7.22	17.47 ± 25.32	0.025

**(c):** 

	High degree of diffuse CD16^+^ infiltrates (≥5 cells)	Low degree of diffuse CD16^+^ infiltrates (<5 cells)	*p* value
Classic (CD14^++^CD16^−^)	496.35 ± 443.38	543.71 ± 368.67	0.627
Intermediate (CD14^++^CD16^−^)	9.65 ± 7.35	17.06 ± 18.79	0.198
Nonclassic (CD14^+^CD16^++^)	3.95 ± 2.32	11.70 ± 10.37	0.001

Severe LN was defined as either Class III or Class IV LN.

## Abbreviations

DC: Dendritic cells
IFN*γ*: Interferon gamma
LN: Lupus nephritis
LPS: Lipopolysaccharide
Mo: Monocytes
SLE: Systemic lupus erythematosus
SLEDAI: Systemic Lupus Erythematosus Disease Activity Index.
